# East Indian Quinine

**Published:** 1867-02

**Authors:** 


*East Indian Quinine.*—The efforts made by the Indian Government to introduce the Cinchona plant in India and Ceylon are detailed in a voluminous blue book lately presented to Parliament. It contains no less than ninety-four reports and letters respecting the efforts made to extend the cultivation of this valuable plant on the Neilgherry Hills, in Wynaad, Coorg, and Travancore, with a goodly number of reports, showing that the ground has been laid for cinchona cultivation in Sikkim, the

Punjaub, Bombay, and Ceylon. There is also a very interesting journal by Mr. Cross, who was commissioned by the Indian Government to make a collection of seeds from the cinchona forests, near Popayan, in South America.

It appears that in 1861 the Government of Madras desired the Home Government to have an analysis made of the East Indian bark, and a number of specimens were collected and sent over by Mr. McIvor, the superintendent of the Government plantations. Mr. John Elliott Howard, the analyst, in his report, stated, "I have great pleasure in informing you that the result of my examination of the bark of *C. succirubra*, grown in India, is very satisfactory. I have thus far only operated upon 500 grains, proceeding cautiously, as the quantity of bark sent is small. I find exactly the same constituents as in South American "red bark," and was able to obtain a first and second crystallization of very white sulphate of quinine mixed (as is usual when obtained from red bark) with sulphate of cinchonidine; I have also obtained some cinchonine. This must be considered very satisfactory, and a promising result, when the immature age of the bark is considered," (viz., two years' growth.) On this favorable report the superintendent was authorized to sell 100,000 plants, which were all speedily applied for by the native planters. A second collection of samples was sent to Mr. Howard for his report, which was still more favorable. He wrote:—

"I have since devoted most careful attention to ascertain by experiment the probable market value, especially of the first two samples of bark sent. It will not be necessary for me to detail the various means by which I succeeded in convincing myself, not only of the existence of the alkaloids, but of their being extant in such a state of purity as is certainly not found in the ordinary samples of red bark imported from South America. The result of my examination tended to show distinctly that cultivation has improved the produce of at least this species of cinchona.

"I must remark that the commercial value of specimens of bark intended for the manufacture of sulphate of quinine can never be ascertained by the mere knowledge of the percentage of alkaloid soluble in ether, since it is necessary that this should be shown to exist in such a state as to crystalize with acids into the required compounds.

"In this case of No. 1, the bark from the thickest part of the lower branches of a *C. succirubra*, two years and five months old, this examination was most satisfactory, confirming that which I stated in my first report as to the facility with which the alkaloids were obtained in a state of purity, although the red coloring matter in the bark is very great. The amount of purified alkaloids I estimated at 6 per cent., consisting of quinine 3·14, cinchonidine 2·06, cinchonine 0·80. This large product of alkaloids might probably be still further increased by surrounding the stem with moss, in the manner which Mr. McIvor has so happily suggested, since Dr. De Vry found 8·409 per cent. of alkaloids in a stem which had been so treated. It seems to me, from this trial, that the East Indian bark, the produce of *C. succirubra*, will rival in price the Bolivian *Calisaya*, which is by no means the case with the bark of the branches of *C. succirubra*, as grown in South America. It is important to remark, that the very high price of between 8s. and 9s., which has quite recently been paid for red bark in this market, applies only to those pieces of bark from the trunk of the tree which possess, from their age, a peculiar bright red appearance. I have forwarded a small vial with commercial sulphate of quinine obtained from this No. 1, as also sulphate of cinchonidine separate from the above. I have only to remark further on this bark, that its appearance bespeaks its good quality, and that there can be no doubt the season chosen (24th of February) is most favorable to its being well secured."

Mr. McIvor, the superintendent of the plantations, appears to have tried the plan of mossing the bark of the plant in order to increase the deposit of the quinine therein, and wished to secure it to himself by patent, but the Government were of opinion that as it was invented in the course of his official duty, it would be a bad precedent to adopt. The experiments made by Mr. Clements to Markham proved, however, that the plan was extremely beneficial; he states that a tree two and a-half years old yielded alkaloids of 2·43 per cent., but 5·20 when mossed for a year. These results, he states, gives us the certainty that the correct method of treating the cinchona trees is to cover the stems with moss, to remove the bark periodically, to renew the bark by mossing the stem, and to allow the tree to continue growing until it attains its utmost size. Dr. De Vry told Mr. Markham that with muriatic acid and caustic soda he treated the green bark and produced a fine powder, consisting of all the febrifuge alkaloids of the bark, which will practically be as efficacious as the expensive sulphate of quinine.

From a return included in the report, it appears that the number of plants on the Neilgherry Hills, which at the beginning of 1863 were a little over 100,000, in May of the present year exceeded 1,100,000. In the other districts mentioned in the list the same activity is manifested. From Ceylon, Mr. Markham reports that the coffee growers have taken to the cultivation of the cinchona in a hearty manner, as many as fifty planters having applied for plants, of which 180,000 have been distributed, the manager of a large estate belonging to Rothschild being the foremost amongst them.

It also appears that Government have ordered new roads to be made especially for the use of the districts in India where this plant is being cultivated, and there can be little doubt that the supply will be greatly increased, as the cultivation of the plant is rapidly extending.—*Chemist and Druggist.*

